# Canada’s Ministerial Order for Reliance on decisions by foreign regulatory agencies for certain drugs: will it benefit pediatric patients?

**DOI:** 10.3389/jpps.2026.17612

**Published:** 2026-10-26

**Authors:** Catherine Y. Lau

**Affiliations:** Independent, Consultant, Toronto, ON, Canada

**Keywords:** Canada, medications, ministerial order, pediatric, reliance, pediatric formulations

## Abstract

The lack of pediatric indications and age-appropriate formulations in Canada has been a major concern for the pediatric community for decades. Although small patient populations and onerous approval processes may deter drug manufacturers from developing or marketing these drugs and formulations, the lack of adequate incentives may also contribute to the problem. In July, 2026, Canada issued a Ministerial Order allowing certain drugs to be approved by relying on reviews conducted by foreign regulatory agencies, with pediatric drugs selected as the first pilot category. Reviews of literature, media publications, guidance documents, government, particularly regulatory documents and product information were conducted to explore Canada’s historical record of pediatric approvals with comparison to US and EU. Problems associated with lack of pediatric medications in Canada were identified. The Ministerial Order of Reliance together with the guidance document were examined to determine whether the proposed reform will solve the pediatric medication crisis in Canada. The Reliance Order may help to alleviate some regulatory nuance and speed up approval times, however, is this sufficient to compel sponsors to submit with no mandatory requirements or added incentives under the current proposal. The submission fee and labour involved probably will outweigh commercial return in most cases, thus not an attractive option for sponsors, leading to unsuccessful outcomes. The manuscript attempts to identify gaps and provide recommendations to ensure the amended policy and new guidance will be enabling to achieve the desired outcomes.

## Introduction

The availability of pediatric drugs and age-appropriate formulations has been a longstanding concern in Canada, and the issue has become more visible with the growing number of new therapies approved for adult indications [1, 2]. Earlier diagnosis and the emergence of novel treatments, including targeted and gene therapies for rare diseases with childhood manifestations, have expanded therapeutic possibilities for pediatric patients. Proactive regulatory agencies such as the US Food and Drug Administration (FDA) and the European Medicines Agency (EMA) have supported pediatric drug development through longstanding mandatory and incentive-based frameworks [3]. Canada, by contrast, continues to face limited approved pediatric indications, a shortage of age-appropriate formulations, quality-control concerns and inconsistencies associated with locally compounded products, and fragmented reimbursement for pediatric medicines [4–6]. This manuscript examines the factors contributing to these challenges, assesses the Government of Canada’s attempt to address the gap through reliance on foreign regulatory decisions, and proposes Canada-specific solutions to alleviate critical pediatric treatment shortages.

## Existing gaps between Canada and other developed countries in pediatric treatment

A review of pediatric clinical literature published over the past decade and indexed in PubMed and Google Scholar indicates that many pediatric researchers have documented the shortage of approved pediatric medications in Canada [1, 5, 6]. Prominent pediatric researchers have emphasized that a central reason for this gap is the absence of mandatory requirements compelling sponsors to conduct pediatric studies or submit the necessary data to Health Canada to obtain pediatric indications, as is required in the United States and the European Union [7]. In addition, Canada’s comparatively modest incentive—six months of data protection when a pediatric application is submitted within 5 years of first approval—has generally not been sufficient to encourage sponsor participation [8].

As shown in Table 1, both the US FDA and EMA have mandatory regulatory requirements for pediatric submissions. The FDA evaluates potential pediatric uses at the time of new drug approval and communicates study expectations to sponsors through a Written Request tied to voluntary incentives via the Best Pharmaceuticals for Children Act (BPCA). Sponsors then prepare a mandatory Pediatric Study Plan (PSP) outlining the studies needed to satisfy FDA requirements. These obligations are paired with substantial incentives, including 6 months of market exclusivity and, more recently, special voucher programs that have been reported to be highly valuable to companies [13]. The European Union initiates this process earlier in development and requires companies to prepare a Pediatric Investigation Plan (PIP), which sets out the pediatric studies to be conducted and must be assessed and approved by the Paediatric Committee (PDCO) [19]. Incentives in the EU are also significant, including 6 months extension of supplementary protection, fee waivers, and up to 10 years of market protection for certain off-patent medicines. In both jurisdictions, a “stick-and-carrot” approach combines enforceable obligations with meaningful rewards to compel the generation and submission of appropriate pediatric data.

**TABLE 1:** Highlights key differences in regulatory requirements and incentives among the United States, the European Union, and Canada.

Feature	United States Food and drug administration (FDA)	European union European medicine agency (EMA)	Canada Health Canada
Approval framework	Mandatory: Pediatric research equity Act (PREA) requires pediatric studies for new drugs sent to companies by FDA using a written request [9]	Mandatory: Pediatric regulation requires a pediatric investigation plan (PIP) for new Medicines. EMA initiates the PIP during early development of the new drug [10]	Voluntary/Developing: No mandatory requirement. A pediatric submission guidance published in 2024. Uses a “national priority list of pediatric drugs” to identify gaps and facilitate access [11]
Incentive program	Voluntary “pull” incentive: Best pharmaceuticals for children Act (BPCA) offers +6 months of marketing exclusivity for completing requested studies. More recently, a voucher program is added [12, 13]	Voluntary “pull” incentive: +6 months of patent protection (SPC extension) for completing an approved PIP. Other incentives include fee waivers, 10-year market protection for off-label drugs. Also fee waivers are available for pediatric drug submissions [14]	Six months data protection added to 8 years data protection for any new chemical entities. Submission of pediatric indication has to be done within 5 years of drug first approval. No incentive for old drugs already on market or lacking pediatric formulation [15]
Key mechanism	Pediatric study plan (PSP): Must be submitted by end of phase 2; can request deferrals/waivers at NDA filing [13]	Paediatric investigation plan (PIP): Must be agreed upon by companies early in development (new proposals may require earlier submission)	National priority list: Identifies drugs approved elsewhere but not in Canada to help prioritize and encourage submissions. A committee of experts monitoring the list [16]
Enforcement	Strong: FDA can require studies under PREA; non-compliance affects approval of new drug submission [17] and delay of review	Strong: EMA requires PIP agreement for market authorisation application (MAA) early in the development phase; non-compliance can lead to penalties, delayed filing and potential public listing [18]	Weak/Lagging: Health Canada cannot compel companies to generate pediatric data or update labels, leading to widespread “off-label” use. Also lack of formulations created local compounding of medication [1, 5]

In Canada, multiple publications by pediatric experts have documented the extent of the problem, including the absence of approved pediatric indications for key medications and the resulting reliance on off-label use, which is not always supported by robust evidence. The shortage of age-appropriate formulations has also contributed to widespread local compounding to meet pediatric needs. However, variable quality control in compounded preparations can compromise both efficacy and safety. More recently, Health Canada, in collaboration with pediatric clinicians across the country, established a Pediatric Advisory Committee and a National Priority List of Pediatric Drugs [16]. In 2024, Health Canada published guidance advising sponsors to submit pediatric data packages, including PSPs from the United States or PIPs from Europe, where available [20]. The Canadian Drug Agency (CDA-AMC), the health technology agency of Canada responsible for reimbursement, also published a December 2025 health technology review, “Federal Policies and Public Reimbursement of Pediatric Medicines in Canada,” which summarized Government of Canada initiatives to improve access to pediatric medicines and reviewed reimbursement policies across provinces [21]. The review included an environmental scan and acknowledged the lack of pediatric indications, shortages of appropriate formulations, and reliance on off-label use. However, it attributed these challenges primarily to small patient populations, low disease prevalence, and difficulties conducting pediatric clinical trials. This explanation is incomplete: the United States and European Union have already generated much of the relevant clinical evidence, and pediatric indications have been granted in those jurisdictions as well as in other mid-sized countries comparable to Canada in population size and gross domestic product. Notably a number of products, Belimumab, Tofacitinib, Abatacept and Anakinra demonstrated adequate pediatric efficacy and safety and sufficient data to support approvals of indication in US did not get HC approvals for either the label or the formulation [3]. Notably, a 2025 publication on tofacitinib summarized positive patient-report-outcome of a post-hoc analysis of a randomized phase 3 study with juvenile idiopathic arthritis [22]; however, a pediatric-friendly liquid formulation remains unavailable in Canada. The Regulatory Decision Summary for a pediatric indication in 2026 [23] mentioned that the company decided not to develop the liquid formulation in Canada due to business reason and not for safety. The lack of commercialization opportunity in Canada and with no added incentive, companies are unwilling to bring pediatric friendly formulation to Canada.

## Ministerial Order of Reliance (MOR)

On 20 December 2025, the Government of Canada, via its official portal, published in the Canada Gazette, Part I, the proposed “Order Providing for Reliance on Decisions of, or Documents Produced by, Foreign Regulatory Authorities in Respect of Certain Drug Classes,” [24] followed by publication in the Canada Gazette, Part II on February 28, following by bringing the regulation into force in 15 July 2026 [25]. The Order is intended to make Health Canada’s review process more efficient by allowing reliance on decisions made, and documents produced, by comparable foreign regulatory authorities. As a pilot initiative, the reliance pathway will apply only to specified drug classes. Because eligibility is limited to selected drug categories, the Order includes an incorporated-by-reference (IBR) list for human and veterinary drugs. For human drugs, the list applies to new drug submissions (NDS), supplemental new drug submissions (SNDS), and abbreviated new drug submissions (ANDS). Submissions may proceed through three mechanisms: general deeming for NDS and ANDS, 120-day filing for NDS, and joint filing for NDS. Similar rules apply to supplemental submissions: both SNDS and supplemental abbreviated new drug submissions (SANDS) may qualify for general deeming, while only SNDS are eligible for 120-day filing and joint filing. The Order identifies potential benefits, including encouraging manufacturers to bring certain drugs to Canada and to file earlier; however, it does not provide additional incentives. The first category under this framework will be pediatric drugs and age-appropriate pediatric formulations. The incorporated-by-reference (IBR) list sets out the eligible drug categories, qualifying criteria, and reference regulatory authorities available to manufacturers.

The list posted in the guidance document, despite the complexity in qualifier description and restrictions in therapeutic classes, such as cell and gene therapies not allowed, some of the critical life-saving therapies for rare diseases in children; follow partially recommendations in the National Priority drug list for pediatric patients. The announcement sounds like a miracle coming true for pediatric patients in Canada, is it time to celebrate or cautiously navigate the Ministerial Order and the accompanying guidance document to identify gaps and challenges that will potentially impact the execution.

## What is missing from the MOR: mandatory regulatory requirements and meaningful incentives

Pediatric regulatory programs in both the United States and Europe have been widely regarded as major successes (Table 1). In the United States, the mandatory PREA framework enables the FDA to require sponsors to conduct pediatric studies, while the voluntary BPCA program provides a complementary “pull” mechanism through financial incentives that can be attractive to sponsors. Publications from 2000 to 2020 have reported that nearly 70% of drugs approved in the United States include pediatric indications or labeling [3]. Although most researchers and clinicians view this progress positively, some have raised concerns that the commercial value of these incentives may, in certain cases, exceed the original intent of the policy. In Europe, pediatric development is addressed even earlier, through mandatory Pediatric Investigation Plans (PIP) for new medicines, supported by market-exclusivity incentives that extend not only to new products but also to certain off-patent medicines. While companies generally recognize the importance of developing safe and effective pediatric indications and age-appropriate formulations, limited commercial value remains a significant deterrent. The United States and European Union have demonstrated the effectiveness of a combined “stick-and-carrot” approach; Canada, by contrast, has not adopted comparable mandatory requirements or incentives and remains a notable laggard among developed jurisdictions.

Canada’s longstanding lag in pediatric regulatory policy and clinical access has been a prominent concern for years, and pediatricians and patient advocacy groups have repeatedly called attention to its consequences. Since 2020, Health Canada has taken several steps to respond, including launching a Pediatric Drug Action Plan that underwent extensive consultation; establishing the Pediatric External Reference Group, co-chaired by Dr. Charlotte Hepburn and Dr. Catherine Litalien, to advise on pediatric drug priorities; and developing a National Priority List of Pediatric Drugs [16]. On February 3, 2021, as part of the MICYRN series, a workshop was convened to develop a roadmap for pediatric medicines in Canada. Key recommendations included establishing a federal pediatric expert committee to support national harmonization and priority setting, accepting trusted foreign regulatory decisions, and introducing additional incentives to encourage commercialization [26]. Health Canada subsequently published a Pediatric Development Plan, promoted voluntary submission of U.S. Pediatric Study Plans or European Pediatric Investigation Plans [27, 28], and issued guidance on pediatric submissions [15, 20]. To date, however, these initiatives do not appear to have generated meaningful increases in pediatric submissions. The critical question is therefore whether the Ministerial Order on reliance, with pediatric drugs selected as the first pilot class, can meaningfully address Canada’s pediatric drug shortages. Without mandatory pediatric development-plan submissions comparable to those required in the United States and European Union, fee waivers, greater flexibility in data-protection timelines, exclusivity for off-label medicines, or a predictable reimbursement pathway, it remains uncertain whether the Order alone will be sufficient to bring more life-saving pediatric medicines to Canadian patients.

## Discussion

Pediatric regulations in the United States, commonly referred to as the “Pediatric Rule,” establish a regulatory mandate requiring the generation of pediatric efficacy and safety data for all new drug submissions when pediatric use can be reasonably anticipated [29]. Both the United States and the European Union have implemented regulatory frameworks that support and enforce pediatric drug development requirements. Together, these frameworks have led to substantial gains: more than 1,000 pediatric indications were added in the United States between 1998 and 2022 [30], and more than 260 were added in the European Union between 2007 and 2016 [31]. Canada, by contrast, has fallen behind. Despite genuine multistakeholder efforts, many critical pediatric indications and age-appropriate formulations remain unavailable. A review of Canadian Product Monographs from 2007 to 2016 found that, among 270 approved products, only 75 included pediatric indications, while 125 contained some pediatric information. A review of Health Canada’s Submission Under Review (SUR) database showed that, between 2017 and 2026, 716 submissions were approved and 37 were rejected; only three of the rejected submissions pertained to pediatric patients. Thus, rejections do not account for the low number of pediatric approvals in Canada [32]. This marked difference underscores the need for urgent policy action [1].

The recently enacted Ministerial Order permitting reliance on foreign regulatory reviews selected pediatric drugs and age-appropriate formulations as the first pilot drug class. The accompanying guidance explains that pediatric medicines were chosen because this area presents an urgent need for regulatory reform and offers an opportunity to gain experience with the reliance pathway. However, in its current form, the guidance is unlikely to generate a meaningful increase in sponsor submissions for the reasons outlined in this manuscript. The following recommendations could strengthen the Order, enhance sponsor engagement, and help make Canada a more predictable and attractive jurisdiction for pediatric submissions.

Additional incentives

1. Extend the current 5-year window for submitting pediatric indications while allowing sponsors to retain the additional 6 months of data protection. One of the drug on the National Priority Drug List for pediatric high-risk Hodgkin Lymphoma, Brentuximab vedotin does not have a pediatric indication in Canada [16]. The drug was approved on Feb 1st, 2013, deadline for submission of pediatric data would be Feb, 2018. However pediatric data was not available until 2021 [33], missing the opportunity of a 6-month pediatric data exclusivity.
2. Introduce fee waivers or meaningful fee reductions for pediatric submissions. The current fee for a New Drug Submission (NDS) in Canada is $ 616,593.00 and for Supplemental New Drug Submission (SNDS), a range from $127,668.00 to $ 319,282.00. Any fee waiver or reduction would be positively received.
3. Provide data-protection incentives for older drugs comparable to those available for new drugs when sponsors seek pediatric indications or formulations.
4. Further reduce administrative and evidentiary submission burdens where high-quality foreign regulatory reviews and decisions are available.
5. Assign priority status to pre-submission discussions involving pediatric indications or age-appropriate formulations.
6. Grant priority review status to pediatric indications or formulations that have already received priority status in the United States or European Union.
7. Establish a target for increasing pediatric submissions and assess progress annually.

Additional responsibilities for sponsors

1. Amend the pediatric guidance to make submission of a Canadian equivalent to a U.S. PSP or European PIP mandatory, delayed submission will potentially affect review timelines for the associated new drug submission.
2. Publicly list delayed or failed submissions of required pediatric development plans.
3. Require annual updates to pediatric development plans to ensure continued accountability and transparency.

Regulatory reliance has been successfully implemented by other review agencies. The Australian Reliance Pathway allows Australia’s Therapeutic Goods Administration (TGA) to use assessment reports from Comparable Overseas Regulators (CORs) to shorten approval timelines such as Australia [34] and also regulatory reliance in Asian-Pacific countries has yielded positive outcomes [35].

The availability of Canada-specific pediatric indications and age-appropriate formulations is critically important. Addressing this gap will require urgent, coordinated action by policymakers, regulators, physicians, patient advocacy groups, and manufacturers to remove regulatory, commercial, and practical barriers that continue to impede timely access to essential treatments for pediatric patients.

## Data Availability

The original contributions presented in the study are included in the article/supplementary material, further inquiries can be directed to the corresponding author.
